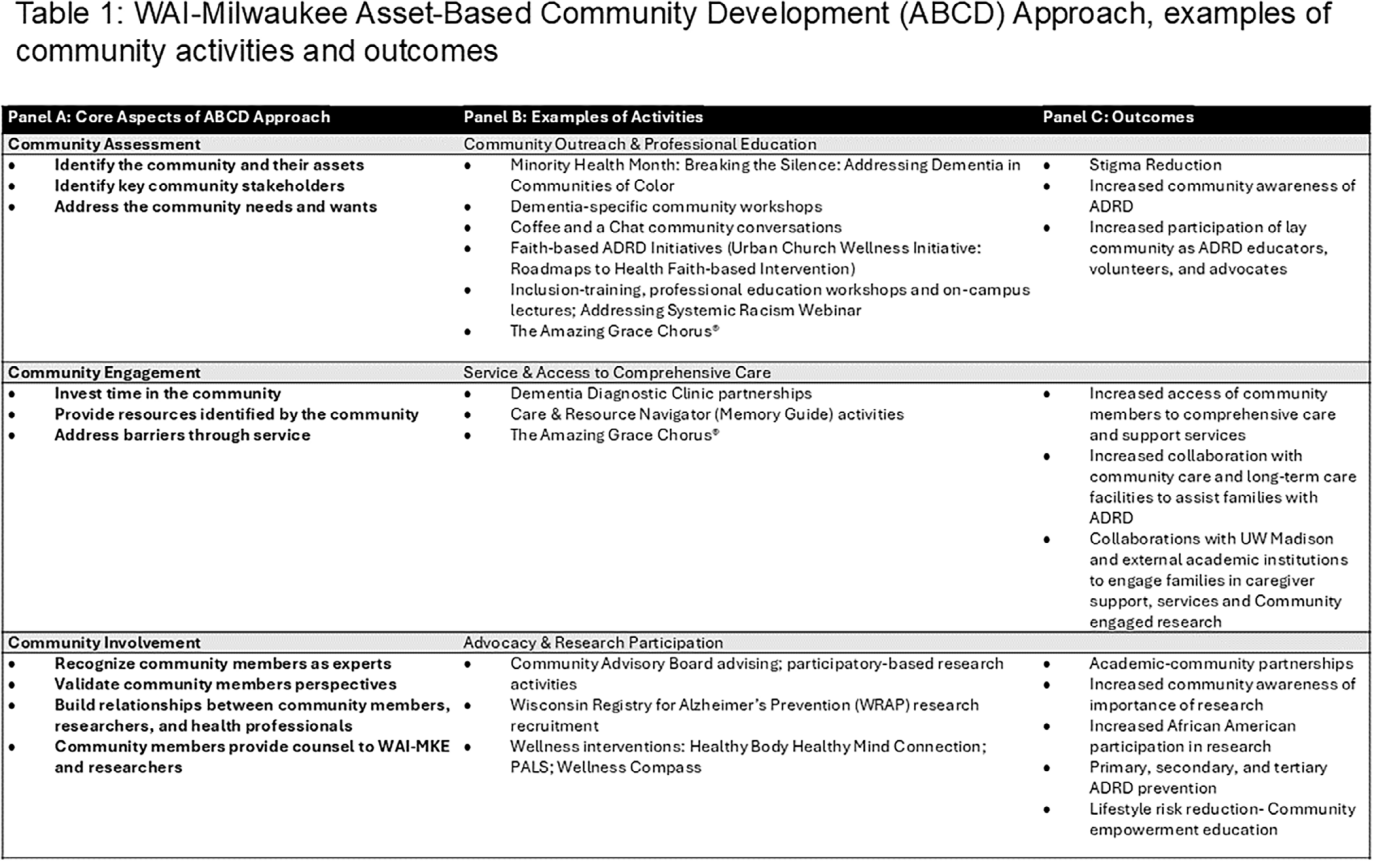# Revisiting Asset‐Based Community Development for African Americans in Research: Long‐Term Impact of Community Engagement in Alzheimer's Disease Studies

**DOI:** 10.1002/alz70858_105900

**Published:** 2025-12-26

**Authors:** Nia C Norris, Stephanie L Houston, Gail D. Morgan, Celena M Ramsey, Maria C Mora Pinzon, Tamara J. LeCaire, Jennifer Landeta‐Vidal, Gina Green‐Harris

**Affiliations:** ^1^ Wisconsin Alzheimer's Institute Regional Milwaukee Office, University of Wisconsin School of Medicine and Public Health, Milwaukee, WI, USA; ^2^ Wisconsin Alzheimer's Institute Regional Milwaukee Office‐ University of Wisconsin School of Medicine and Public Health, Milwaukee, WI, USA; ^3^ Wisconsin Alzheimer's Institute Regional Milwaukee Office, University of Wisconsin‐Madison School of Medicine and Public Health, Milwaukee, WI, USA; ^4^ Department of Medicine, Division of Geriatrics, School of Medicine and Public Health, University of Wisconsin‐Madison, Madison, WI, USA; ^5^ Wisconsin Alzheimer's Institute, University of Wisconsin School of Medicine and Public Health, Madison, WI, USA; ^6^ Wisconsin Alzheimer's Institute, University of Wisconsin School of Medicine and Public Health, Madison, WI, USA

## Abstract

**Background:**

Asset‐based community development (ABCD) approaches were first implemented in 2008 by the Wisconsin Alzheimer's Institute (WAI) Regional Milwaukee Office. These efforts increased social support for African American caregivers and elders living with or at risk for Alzheimer's disease and related dementias (ADRD) and African American representation in research. We describe the reach of activities and the impact that these activities have had since inception.

**Method:**

Various methods of engagement and relationships have been established, including with diagnostic memory clinics, Aging Disability Resource Centers, community‐based organizations, and academic institutions. Data for this evaluation were collected from participant registries, observation, and surveys. Data were summarized following the Asset Based Framework (Community Assessment, Community Engagement, Community Involvement) and descriptive statistics are provided for activities performed and networks established.

**Result:**

Community Assessment activities include hosting community outreach and education workshops, such as ‘Breaking the Silence: Addressing Dementia in Communities of Color’, which served 5,000 people through multi‐level collaborative educational activities (Table 1). Community Engagement activities include those that provide direct services and resources to individuals (Table 1); the WAI Regional Milwaukee Office performed 500 in‐home care visits and memory and health screenings, resulting in 175 diagnoses and connections to health care services. Over 200 families were connected to social services, ultimately allowing people living with dementia to remain at home. Community Involvement includes activities that build relationships with communities and promote participation in research (Table 1). In the first four years of the Wisconsin Registry for Alzheimer's Prevention (WRAP), only six African Americans were enrolled; our efforts increased this number to 301.

**Conclusion:**

Efforts to foster intentional partnerships and lasting collaborations have transformed researchers' perspectives on community‐based research and boosted involvement from historically marginalized groups. Implementation of the ABCD model's core aspects resulted in increased participation by the lay community, increased collaboration with care facilities, and increased African American participation in research. The community's awareness of various health topics demonstrates our impact in bridging knowledge gaps and addressing issues affecting communities of color. These initiatives strengthen our connections and yield tangible benefits for the communities we serve.